# Supplementary material for: Low cost DNA data storage using photolithographic synthesis and advanced information reconstruction and error correction
Source: Nat Commun. 2020 Oct 22;11:5345. doi: 10.1038/s41467-020-19148-3 (PMC7582880; doi:10.1038/s41467-020-19148-3)
Supplement: Supplementary file 2 — Supporting Information [file 41467_2020_19148_MOESM2_ESM.pdf]

Supporting Information

Supplementary Note 1

Encoding: Data to DNA Sequences.

The design of encoder and decoder were jointly developed parsing the structure of errors introduced in the light-directed synthesis. We encoded 99103 bytes on *M* = 16383 fragments having a length of 60 nt, which translates to 0.94 bits/nt. Due to the direct synthesis of all information, this value represents the effective informational density in the oligo pool and is comparable or higher than most of the densities in previous work (see Table 1).

- Randomization: We start by splitting the information to be stored into *K* = 10977 many fragments of length 14 × 6 bits. We then multiply the bits of the information with a pseudorandom sequence. This step is invertible (since we know the pseudorandom sequence). After this randomization step, the bits appear random for statistical purposes.
- Outer-decoding: We consider each fragment $c_i = [c_{i1}, \ldots, c_{i6}], i = 1, \ldots, K,$ as consisting of 6 blocks, $c_{ij}, j = 1, \ldots, 6,$ each block of length 14 bits. Use a Reed-Solomon code of length 16383 with symbols in $\{0, \ldots, 2^{14-1}\}$ to encode $c_{i1}, \ldots, c_{ik}$ to obtain the codeword $c_{i1}, \ldots, c_{in}$ for each i. This yields the new, redundant sequences $c_i = [c_{i1}, \ldots, c_{i6}], i = K + 1, \ldots M$.
- Indexing: Next, we add a unique index of length 24 bits to each of the *M* = 16383 sequences. The sequences c_i now have length $24 + 14 \cdot 6 = 16 \cdot 6$ bits.

- Inner-decoding: We now view each sequence c_i as consisting of 18 symbols of length 6 bits and add two parity symbols by inner-decoding with a Reed-Solomon code with symbols in $\{0, \dots, 2^{6-1}\}$. This yields sequences of length $20 \cdot 6$ bits.
- Mapping to DNA: Finally, we map each sequence of length $20 \cdot 6$ bits to DNA sequences of length 60 nt, simply by using the map $00 \rightarrow \mathrm{A}, 01 \rightarrow \mathrm{C}, 10 \rightarrow \mathrm{G}, 11 \rightarrow \mathrm{T}$.

By randomization of the input, we elegantly circumvent homopolymers that become very unlikely via multiplication of pseudorandom sequences. The negligible increase in reading error rates due to rare homopolymers is more effectively dealt with using our error correcting mechanisms as opposed to actively avoiding them. Additionally, randomization yields sequences that are pairwise close to orthogonal to each other, which is desirable for distinguishing them for example by a clustering algorithm.

Supplementary Note 2

Library preparation with Swift Biosciences commercial kit

To further shed light on the functionality of the commercial kit used to prepare sequencing libraries out of ssDNA, gel electrophoresis pictures in agarose gel of different stages within the preparation were recorded. Due to abundance and better visibility in gel electrophoresis, model DNA of two different lengths were employed and the maximum input amount of 250 ng was taken. For this purpose, a 60mer (Microsynth AG, CH) where each base position is randomly distributed, representing a very similar DNA molecule was synthesized conventionally. Another 158 bp long dsDNA random

library was used as a reference for the comparison of longer starting templates and double stranded inputs. The two DNA samples serve as dummies to investigate how enzymes attach read adapters and to test overall kit robustness and integrity. Supplementary Figure 1 shows the process employed in the preparation of a library[1].

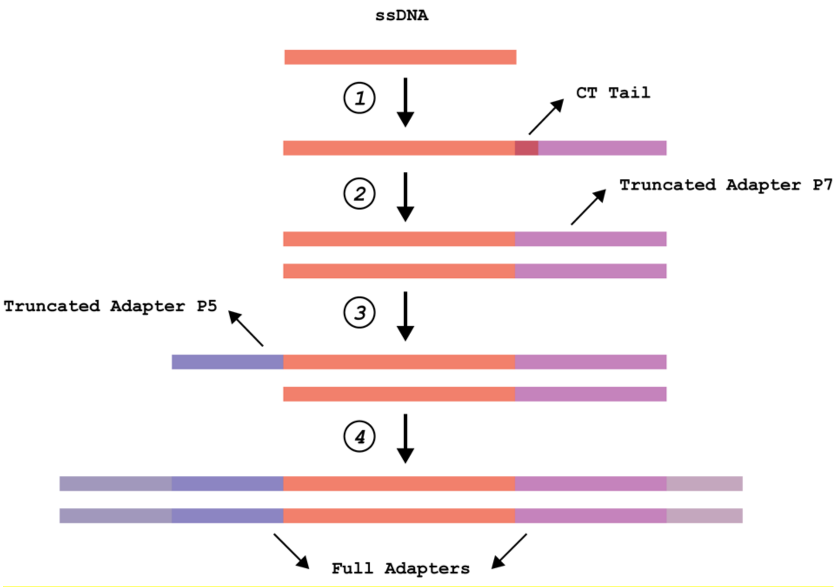

Supplementary Fig. 1: Workflow of the Accel-NGS 1S Plus DNA Library Kit (Swift Biosciences) adapted from the manual. DNA fragments first undergo 3’ ligation of truncated adapter (1) and duplexing (2), followed by 5’ ligation of truncated adapter (3) and finally PCR (4) that concatenates the full-length adapters.

First, the template ssDNA fragments are heated for 2 min to 95 °C and immediately put on ice to ensure only single strands partaking in the first reaction. A truncated adapter is ligated to the 3’ prime end of the template. The reaction mixture essentially consists of a template independent polymerase in the form of terminal deoxynucleotidyl transferase (TdT) which is inhibited in their activity by mainly two factors: a suitable reaction environment controlled by ion concentrations without inhibiting the activity of involved enzymes and an attenuator-adapter complex. TdT adds single nucleotides to the 3’ end

of the template DNA which is directed by the composition of the reaction solution providing primarily deoxycytidine and deoxythymidine monophosphate (dCMP and dTMP). The attenuator part of the inhibiting complex is complementary to the CT tail growing in this manner and additionally stops uncontrolled growth of the tail with a blocking group that prevents more bases from being bound to the template. It further comprises a section adjacent to the attenuator sequence that is complementary to a part of an NGS adapter sequence (Truncated adapter P7, see Supplementary Fig. 1) which is also present in the reaction mixture. Another enzyme concatenates the 3' CT tail end and the truncated adapter sequence in place. Enzymes and chemicals for the second step that is rendering the template-adapter intermediate double stranded, is directly applied to the reaction mixture without purification. The bulk of the reaction takes place at 37 °C (15 min) with a 2 min denaturation step at 95 °C. Supplementary Fig. 2b shows the length of the templates after these first two steps. The templates are now around 100 bp for the 60mer and around 200 bp for the 158mer, revealing a truncated adapter length of ca. 40 bp which is about 2/3 of the full-length adapter. Similarly, the P5 truncated adapter is ligated to the 3' end of the template in the third step allowing the use of PCR primers completing adapter addition and significantly increase the copy number of the template strands (Step 4). Template length after Step 3 is ~130 bp for the 60mer and ~ 230 bp for the 158mer, showing a P5 truncated adapter length of ca. 30 bp (approximately half of the full-length adapter). Especially this step is not straight-forward to see in the gel pictures. The reason for this is probably that the attached adapter part is small compared to the rest of the molecule and single-stranded which likely doesn't change elution properties to an extent that can be easily detected.

The ready-to-sequence product after PCR has a target length of 181 bp for the 60mer and 279 bp for the 158mer. This can be confirmed by gel electrophoresis (see Supplementary Fig. 2d).

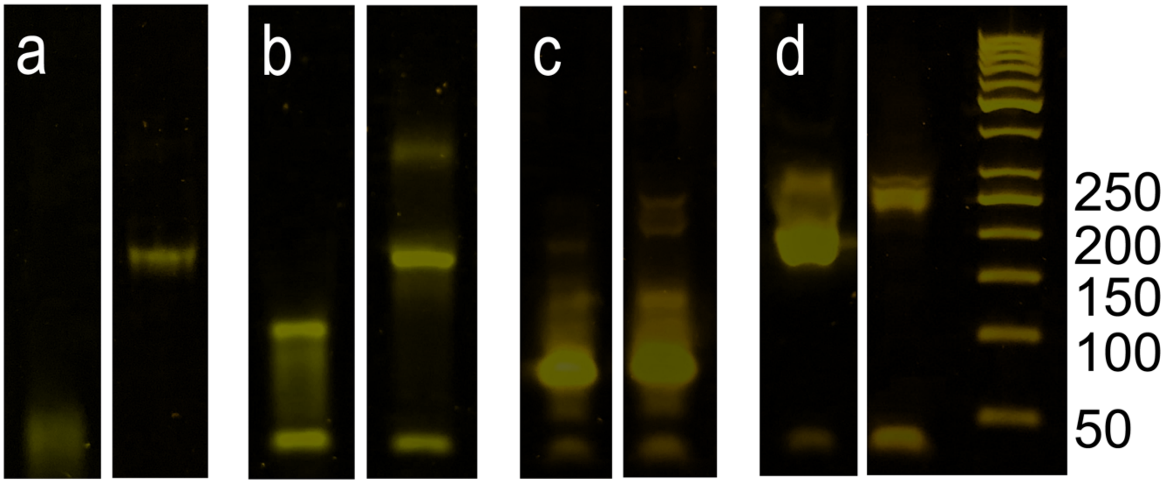

Supplementary Fig. 2: **Agarose gel (2%, Invitrogen E-Gel EX 2%) images**. The left lane always shows the random 60mer oligo, the middle lane shows the 158mer double-stranded library and the right lane shows a 50 bp DNA ladder (Thermo Scientific GeneRuler) as a reference. **a** Original input **b** Fragments after step 2 of the library preparation. **c** Fragments after step 3 of the library preparation. **d** Final product after PCR, ready-to-sequence. The images are compiled from several different gel electrophoresis runs. The series of experiments **a** – **d** were repeated five times in similar experimental set-ups showing comparable results. Source data are provided as a Source Data file.

Supplementary Note 3

Clustering of Sequences

The main challenge in clustering the data is that we typically have millions of sequences, from which we generate potentially millions of clusters, which is computationally very expensive when using standard methods for clustering. In particular, clustering methods that compute pairwise distances are infeasible. A by now standard algorithmic approach

for clustering large collections of data efficiently is locality sensitive hashing (LSH)[2]. A hashing based approach for clustering sequences in the context of DNA storage was first proposed by Rashtchian *et al*.[3]. The work by Rashtchian *et al*. uses a slightly different hash function than the min-hash function we use, but the main difference is that the algorithm by Rashtchian *et al*. partitions the noisy reads into clusters via an iterative approach, whereas our approach is sequential and only requires one pass through the data. Moreover, the algorithm by Rashtchian *et al*. requires the length of the reads to be sufficiently long, whereas we have very short reads.

In this section, we study two efficient clustering approaches: First, a naïve and very fast clustering algorithms that clusters sequences by the beginning of each sequence, and second, a variant of the LSH approach from Havelivala *et al*.[2], which was originally designed for clustering web documents.

LSH based clustering

Clustering algorithms are based on a notion of distance between DNA sequences or strings. The natural measure of distance between two sequences — given that the perturbations are deletions, insertions, and substitutions — is the edit distance. The edit distance between two sequences is the minimal number of deletions, insertions, and substitutions required to transform one sequence into the other. However, the edit distance is expensive to compute.

The clustering method we propose for clustering the noisy reads is based on locality sensitive hashing (LSH), and is inspired by an algorithm originally proposed for clustering web documents[2]. LSH relies on a cheaper-to-compute proxy for the edit distance, computed using the so-called Min-Hashing (MH) method.

To compute a proxy for the edit distance, we first split each sequence into overlapping sub-sequences of length *k*, called *k*-mers (also called shingles of length *k* or q-grams). For example, for *k* = 2, the sequence ACGT becomes the set {AC,CG,GT}. Next, we assign a unique number to each *k*-mer. For example, view A, C, T and G as 0, 1, 2, and 3, respectively, and allocate a power of 4 to each position. Then, given the *k*-mer ACCT, the assigned number would be $4^0.0 + 4^1.1 + 4^2.1 + 4^3.2 = 148$. Hence, we obtain a set of numbers for each sequence.

Now, each sequence is represented by a set of numbers and similarity between the two sets can be measured by the Jaccard similarity of two sets $\mathcal{A}, \mathcal{B}$, defined as:

$$J(\mathcal{A}, \mathcal{B}) = \frac{|\mathcal{A} \cap \mathcal{B}|}{|\mathcal{A} \cup \mathcal{B}|}, \quad (1)$$

where $|\mathcal{A}|$ denotes the cardinality of the set $\mathcal{A}$, i.e., the number of elements in the set. The clustering method we propose does not compute the Jaccard distance for all pairs of sequences, since this is computationally not feasible. Instead, the MH method generates a number of signatures for each sequence (specifically, from each set of numbers), and assigns two sequences to the same cluster if sufficiently many of the signatures are the same.

MH-signatures. MH signatures are generated as follows, from the sets of numbers corresponding to the *k*-mers of the sequences. We first generate permutations π of $\{0, \dots, N-1\}$, where we assume that 0 and *N* - 1 are the minimum and maximum value an element of the set can take on. The *i*-th element of the MH signature of the set $\mathcal{A}$, denoted by $h_\pi(\mathcal{A})$, is then the minimum of the permutation applied to the set, i.e.,

$h_\pi(\mathcal{A}) = \min(\pi(j), j \in \mathcal{A})$. For example, consider a set $\mathcal{A} = \{1,2,3,4\}$ and a random permutation $\pi = \{6,2,3,1,8,5,0,4\}$. Then

$$h_\pi(\mathcal{A}) = \min(6,2,3,1) = 1.$$

MH signatures have the property that in expectation over the random permutation π,

$$\mathbb{E}[h_\pi(\mathcal{A}) = h_\pi(\mathcal{B})] = J(\mathcal{A}, \mathcal{B}),$$

thus computing many such MH signatures enables estimating the Jaccard similarity, see Broder *et al.*[4] for further details. Stated differently, two sets with Jaccard similarity *p* have the same MH signature with probability *p*.

Extracting similar pairs of sequences. The clustering algorithm is based on extracting similar pairs which in turn is based on generating so-called locality sensitive hashing (LSH) signatures for each sequence and clustering sequences together if LSH signatures of a pair are the same. An LSH signature consists of ℓ_{LSH} many MH signatures (we discuss the choice of ℓ_{LSH} later).

The algorithm repeats the following steps ℓ_{LSH}-many times to generate a set of pairs:

1. Generate k_{LSH} many permutations and for each DNA sequence indexed by *j*, converted to the set $\mathcal{A}_j$, generate a LSH signature, defined as

$$sig(\mathcal{A}_j) = \left(h_{\pi_1}(\mathcal{A}_j), \ldots, h_{\pi_{k_{LSH}}}(\mathcal{A}_j)\right).$$

2. Sort all sequences by their LSH signatures, and for each set of matching LSH signatures, add all pairs to the set of pairs.

The rationale behind this approach is that if k_{LSH} is sufficiently large, then only sequences that are very similar will end up as similar pairs. Smaller k_{LSH} make it more likely that less similar sequences are also paired together. Note that the process is randomized due to

the random min hash sequences; repeating the pairing process ℓ_{LSH} many times makes sure that sequences that are very similar are also paired together.

Filtering the pairs. As a next step, we go through all the pairs and after performing a local pairwise alignment on each pair, we drop the pair from the set if the number of matched characters falls below a threshold (we set the threshold to 30).

Generating clusters from similar pairs. Finally, we generate clusters from the pairs by first sorting the pairs. Note that each pair appears twice, once as (u, v) and once as (v, u). We sequentially go through all pairs, and the first time that node u appears in the scan, it is marked as a cluster center. All subsequent nodes v that appear in pairs of the form (u, v) are marked as belonging to the cluster of u and are not considered again.

Choice of parameters

The clustering algorithm has four tuning or hyper parameters: the size of the *k*-mers, *k*, the number of MH signatures to be concatenated in order to obtain a LSH signature, k_{LSH}, and the number of LSH signatures to be extracted, ℓ_{LSH}.

According to Havelivala *et al.*[2] setting *k* to 3 or 4 works well for clustering documents. As discussed next, for our application of clustering DNA sequences, which belongs to a smaller alphabet than text, larger values of *k* work better. If *k* is too small, then sequences that are not similar can be very similar according to the Jaccard similarity (take *k* = 1 as an extreme case), and if *k* is too large, then sequences that are close in edit distance are not similar. Our goal is to choose *k* such that sequences originating from the same original

sequence are similar in Jaccard distance and sequences originating from different sequences are not similar.

To determine a good choice for *k* based on our data, we first select a pair of ground-truth clusters by taking two random original sequences and performing an exhaustive search through all noisy reads. We keep the reads with edit distance less than 10 to the respective original sequences. Then, for *k* varying from 3 to 8, we calculate the fraction of pairs of sequences with the first and second sequence belonging to the first and second cluster, of Jaccard similarity smaller than 0.05, as this corresponds to dissimilar sequences in Jaccard similarity. The results, averaged over 190 pairs of random cluster centers, plotted in Supplementary Fig. 3, show that for $k \geq 6$, with probability close to one, sequences belonging to two different clusters have Jaccard similarity at most 0.05.

Next, we consider the in-cluster similarity of the sequences for *k* varying from 3 to 8. Specifically, we computed the average mean Jaccard similarity between all pairs in a cluster, and averaged over 100 randomly-chosen ground-truth clusters.

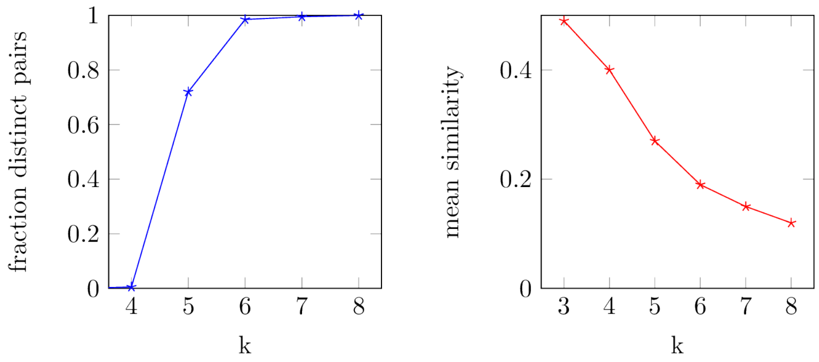

Supplementary Fig. 3: Choice of parameter *k* on distinct pairs and in-cluster similarity. Left: fraction of pairs from different clusters with Jaccard similarity smaller than 0.05 as a function of the *k*-mer size. Right: mean Jaccard similarity as a function of the *k*-mer size. Source data are provided as a Source Data file.

Based on the results from Supplementary Fig. 3, k = 7 is a good choice as it ensures that the sequences from different clusters are sufficiently distinct in Jaccard similarity, while at the same time the sequences from the same cluster are close in Jaccard similarity.

As for the length of the LSH signature, we set k_{LSH} = 3, since choosing this parameter too large or too small results in occurrence of false positives and false negatives.

Finally, the parameter ℓ_{LSH} is chosen based on the desired degree of similarity for assigning similar sequences to the same cluster. Specifically, our goal is to assign similar sequences to the same cluster if the corresponding Jaccard similarity exceeds 0.5. If two sequences have Jaccard similarity 0.5, then the probability of them having the same MH signature is 0.5. Since we concatenate k_{LSH} MH signatures to obtain one LSH signature, the probability that the mentioned sequences have the same LSH signature is $0.5^{k_{LSH}}$. Therefore, we need to extract at least $\ell_{LSH} = \frac{1}{0.5^{k_{LSH}}} = 8$. LSH signatures to ensure that similar pairs are extracted with high probability.

Clustering performance

In this section we compare the clustering performance to a trivial clustering method that only clusters based on the first 15 characters of the sequences (we picked 15 as it did yield the best performance) and assign sequences to the same cluster if the first 15 characters are the same. The rationale behind this method is that the beginning of each sequence has fewer errors than the rest.

We compare performance in terms of the fraction of recovered sequences, which is defined as the fraction of original sequences that appear at least once in the candidates generated by clustering followed by multiple alignment and majority voting, as described

in the main body of this paper. A visualization of this process can be observed in Supplementary Fig. 4. Supplementary Table 1 shows that the fraction of recovered sequences is about 5% larger with the locality sensitive hashing-based clustering algorithm. The runtime of the LSH method is by about a factor of eight larger than that of the trivial clustering method. Note that the most expensive step by far is the alignment of the clusters which is larger for the LSH based clustering algorithm since it generates more candidate clusters, with our choice of parameters.

Supplementary Table 1: Performance of fraction of recovered sequences.

Algorithm	Fraction recovered (%)	Errors	Erasures
LSH	79.20	6657	41
LSH + Filtering	83.71	5676	68
TC	74.74	7687	264

In order to check whether removing outliers, if any, from the clusters generated by the LSH method enhances the fraction of recovered sequences, we calculated the mean edit distance of each cluster element from the rest of the elements. Afterwards, we dropped the ones with mean edit distance less than a threshold (24 was the optimum threshold). We observed that the results almost remained the same. Thus, we concluded that either another method for outlier removal needs to be employed or the amount of outliers within each cluster is negligible.

```
Reads     5'-GTGT-A-C-GGTGT--TAAGG...-3'
             GTGTTA-CGGGTGTGCTAAGG
             GTGTTA--TGGTG--CTAAGG
             GTGTTA--CGGCGTGTCAAGG
             GTGTTATCGGGTGTCTCAAGG
             GTGTTA-CGGGTGATCTAAGG
             GTGTGA-CGGGTGT-CTAAGA
             GTGTTA-CGGGTGT-CTAAGT
             GTGTTA-CGGGTG--ACTAGG
             --GTTA--CGGT-T-CTAAGG
             GTGTTA-CCGGTGTGCTAAGG
             GTGTTA-CGGGTGT-CTAAGG
             GTGTTACAGGGT---CTAAGG
             GTGTTA-CGGGTGT-CTAAGG
             GTGTAC--GGGTGT-CTAAGG
             _____________________
A-counts   0  0  0  0  1 14  0  1  0  0  0  0  0  1  0  1  0 14 15  0  1
C-counts   0  0  0  0  0  1  1 10  3  0  0  1  0  0  1 12  3  0  0  0  0
G-counts  14  0 15  0  0  0  0  0 10 15 15  0 13 11  3  0  0  0  0 15 13
T-counts   0 14  0 15 14  0  1  0  1  0  0 14  0  0  1  2 12  1  0  0  1
--counts   1  1  0  0  1  0 13  4  1  0  0  0  2  3 10  0  0  0  0  0  0
             =====================
Vote         GTGTTA-CGGGTGT-CTAAGG
Original     GTGTTA CGGGTGT CTAAGG
```

Supplementary Fig. 4: Alignment of a cluster. The indicated colors are insertion (blue), deletion (red) and substitution errors (green). The majority voting is visualized by the distinct counts for every nucleotide and the comparison to the original sequence.

Supplementary Note 4

Overview of DNA Array Synthesis methods

Agilent. The idea to employ commercial ink-jet print heads, as they have been used in the printing industry for over 65 years, in DNA synthesis, was first proposed in 1996[5]. The glass wafers used for synthesis are coated with a special mixture of silanes, rendering them hydrophobic with a specific amount of free hydroxy groups for the nucleoside to bind[6]. A similar concept is exploited in the synthesis surface structure in

Twist Biosciences' system. Standard phosphoramidites are delivered with ink-jet pumps which are fabricated with etching techniques well-known in the semiconductor industry. Small cavities etched into silicon wafers are sealed with thin glass plates that can be deformed by applying a current to a piezoelectric element sitting on top of the glass plate. The reduction in volume effects the release of liquid in the picoliter range and enables fast and precise loading of synthesis wells.

Twist Biosciences. The fabrication of both the synthesis chip and delivery system for the technology used at Twist Biosciences, are closely related to semiconductor technology. A 96-well plate, as it is also applied in common liquid handling machines, is further tailored for the needs of error-free and automated synthesis. Each of the wells is comprised of 100 smaller wells, called loci, where oligonucleotide synthesis takes place. The proficient coating of hydrophilic, hydrophobic and mixtures of inert and hydrophilic silanes which can bind to nucleosides, enables the growth of 100-mers with very low-error rate[7]. To increase the yield which is compromised due to the reduced number of binding sites in loci, the surface texture is additionally changed by incorporating protrusions and rivets, thereby extending the synthesis surface. Within the surface energy constraints introduced in etching and coating steps, ink-jet print heads precisely deposit small volumes which are further split by surface forces, covering several loci. The underlying phosphoramidite chemistry featuring acid-labile DMT protecting groups enables average yields of about 99.9%, leading to close to 90% of error-free oligonucleotide strands on a chip.

CustomArray. Complementary metal oxide semiconductors (CMOS) are used to create individually controllable microelectrodes with a high density. Similar to DLP-MAS this

allows parallel addressing and control of individual oligos during the synthesis process[8]. The platinum electrodes which can be switched between several different channels generate localized protons which change the pH confined with an appropriate buffer system. Within this so-called “virtual flask” any form of chemical reaction depending on pH can be steered towards the desired product. The immobilization of the growing oligo chain is realized by a porous layer with a thickness of 1 to 20 µm coated onto the electrode material. This creates three-dimensional, high surface areas for the synthesis to take place which can be tailored also for different applications e.g. various biomolecule synthesis and detection. The delivery of chemicals occurs with the help of a microfluidic device featuring several connected chambers controlled by electrochemical pumps[9].

DLP-MAS. Maskless array synthesis with digital light processing was enabled by the fabrication of the first working digital micromirror device by Texas Instruments (TI)[10]. Originally developed for projection technology, scientist soon used the chips for the production of oligonucleotides. A DMD consists of 786432 (XGA) to 8847350 (4K) micromirrors which can be tilted ±12° individually by applying a small current to the CMOS address circuitry. Light from a UV light source is shaped and directed to the DMD, where every micromirror is set to the appropriate position in order to illuminate the synthesis surfaces. In the “ON” position, light hits the pixel of the corresponding area inside the flow chamber and triggers the deprotection of the 5′ hydroxyl group of the phosphoramidite. The photochemistry applied in this technique relies on photo-labile protecting groups as e.g. 2-(2-nitrophenyl)-propoxycarbonyl (NPPOC) or benzoyl-2-(2-

nitrophenyl)propoxycarbonyl (Bz-NPPOC) which efficiently absorb photons in the UV-A range.

Supplementary Note 5

Calculations for Table 1

- The cost for our synthesis is calculated based on non-industry prices for monomers, reagents and solvents. Storing 0.099103 MB with a cost of 52.54 US$ of total synthesis cost results in approximately 530 US$ MB^{-1}.
- The physical density in this work stems from the input used for the library preparation kit. For the 99103 bytes recovered from 23.7 ng of DNA, a physical density of 4.2 Terrabytes g^{-1} can be determined. With the lowest amount still viable for library preparation, a physical density of 99103 bytes/10 pg = 9.9 PB g^{-1} could have been achieved.
- Input data for Church *et al.*[11], Goldmann *et al.*[12], Grass *et al.*[13] and Erlich *et al.*[14] was taken from Erlich *et al*.
- The net information density (excluding adapter annealing sites) and the physical density is taken from calculations from Erlich *et al.*[14] and Organick *et al.*[15]
- Information density including primers is taken from calculations of Organick *et al.*[15]
- Cost per megabyte: Church *et al.*[11]: For the lack of more precise pricing information of the Agilent oligo synthesis service, the synthesis costs were approximated by linear correlation of prices for 7.5k (2650 US$), 15k (5300 US$), 100k (10070 US$) and 244k (22154 US$) feature oligo pools. The indicated

prices are list prices as of December 2018. Thus, an estimated price of 7570 US$ for a 60k oligo pool with 150 nt length was estimated, resulting in 11650 US$ MB^{-1}. Goldmann *et al.*[12]: As indicated in the SI. Grass *et al.*[13]: Calculated from 2500 US$ CustomArray pool and data encoded. Erlich *et al.*[14]: Calculated from 7000 US$ Twist Biosciences oligo pool and the data encoded. Organick *et al.*[15]: 1’3400’000 features were encoded using the Twist Biosciences oligo pool service. The biggest Twist chip produces 696’000 distinct oligos and has a list price of 59’160 US$ for 150 nt long oligos. With little over 200 MB produced, a price of (19 x 59160 US$)/200.2 MB = 5610 US$ MB^{-1} can be calculated if an approximated value of 19 synthesized pool is taken and no discounts are included.

- Lee *et al.*[16] encoded 96 bits partitioned into 12 x 8 bits excluding a 4 bit address. In total, 144 bits were encoded in 12 strands having a median length of 26 nt. This results in 144 bits/(12*26 nt) = 0.46 bits/nt net information stored. There was not enough information concerning synthesis yield in order to calculate the physical density. The cost for the synthesis could be calculated from the number of features and cycles required, both 12, and the indicated cost of chemicals, if TdT enzyme is reused, 4.38 US$ per mL. Supposing that the employed liquid handler was operated at minimum capacity of 0.2 µL, the total cost for the synthesis were (4.38/1000) US$/(µL * cycle * feature) * 0.2 µL * 12 features * 8 cycles = 0.08 US$ corresponding to approximately 7010 US$ MB^{-1}. It should be noted that for the most recently developed liquid handling system[17], this can be improved 2-3 orders of magnitude.

Supplementary Figures

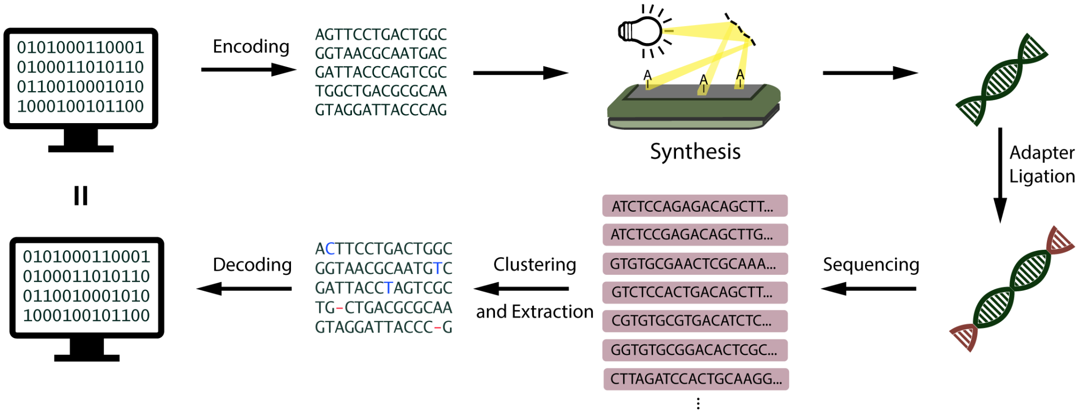

***Supplementary Fig. 5*: Scheme of the information channel**. *Data is encoded to DNA which is produced by light-directed synthesis. Concatenating Illumina Adapters converts the library into a readable file. The reads are clustered, possible candidates extracted and decoded to recover the stored information.*

Supplementary Tables

Supplementary Table 2: Detailed description of consumption and prices used for one synthesis.

Group	*Item*	*Amount*	*Price*	*Unit*	*Cost*
Amidites	*Bz-NPPOC dA*	0.08	81.36	US$ g^{-1}	6.18
	Bz-NPPOC dC	0.05	81.36	US$ g^{-1}	4.07
	Bz-NPPOC dG	0.07	81.36	US$ g^{-1}	6.02
	Bz-NPPOC dT	0.06	81.36	US$ g^{-1}	4.80
	Cleavable dT	0.25	45.20	US$ mL^{-1}	11.30
Solvents & Reagents	*ACN*	175.00	0.02	US$ mL^{-1}	3.29
	Activator	21.00	1.02	US$ mL^{-1}	0.21
	Oxidizer	9.00	0.08	US$ mL^{-1}	0.76
	Exposure solvent	80.00	0.33	US$ g^{-1}	0.53
	β-carotene	0.03	15.86	US$ g^{-1}	0.45
Slide functionalization	*Glass slide*	1.00	1.00	US$ $slide^{-1}$	1.00
	Silanizing reagent	0.66	1.81	US$ g^{-1}	1.19
	Ethanol	90.00	0.01	US$ mL^{-1}	0.54
Library preparation	*ZipTip*	2.00	5.93	US$ tip^{-1}	11.87
	Ethylenediamine	20.00	0.02	US$ mL^{-1}	0.33
				**Total**	**52.54**

Supplementary Table 3: Sequencing Adapters utilized to read encoded data. The underlined bases represent sequencing indeces.

P5 TruSeq LT Adapter	5'AATGATACGGCGACCACCGAGATCTACACTCTTT CCCTACACGACGCTCTTCCGATCT 3'
P7 TruSeq LT Adapter with Index 14	5'GATCGGAAGAGCACACGTCTGAACTCCAGTCAC AGTTCCGTATCTCGTATGCCGTCTTCTGCTTG
P7 TruSeq LT Adapter with Index 16	5'GATCGGAAGAGCACACGTCTGAACTCCAGTCAC CCGTCCCGATCTCGTATGCCGTCTTCTGCTTG
P7 TruSeq LT Adapter with Index 17	5'GATCGGAAGAGCACACGTCTGAACTCCAGTCAC GTCCGCACATCTCGTATGCCGTCTTCTGCTTG

Supplementary Discussion

Cost Comparison of Synthesis Techniques

For a better comparison of costs concerning our method and prices of competitors already established on the market, we performed a rough cost estimation. We chose list prices that do not include academic discounts for all mentioned consumer prices. The data encoded was calculated from the number of features times the net info which was set to 1 bit/nt for all methods. For CustomArray we chose the biggest chip with 92'918 features and a net length of 130 nt. Twist Biosciences' chip with 600'000 features and a net length of 210 has the best trade-off between price and amount of encoded data and in the case of Agilent, a chip with 244'000 features and a net length of 190 nt was

chosen. The net length takes into account 20 nt on 5' and 3' end, needed for sequencing primer ligation. Full adapter ligation as employed in this work is primarily not restricted to photo-directed synthesis. However, for automation purposes, it remains questionable if there is a cost advantage by using it for established synthesis techniques. Here, commonly applied working schemes are used to compare the state-of-the-art. While we cannot guess the machine cost, personnel cost, marketing cost and other costs for the already existing suppliers, we have access to the final sales price, and can compare this with a sales price using a cost model for the maskless DNA synthesis presented here (see Supplementary Table 4).

Supplementary Table 4: Cost estimations for industrial-scale photo-directed synthesis machine in comparison with established suppliers.

	CustomArray	Twist Biosciences	Agilent	Our Work
Machine CAPEX/Synthesis				1333.33
Chemicals				52.54
Personnel				400.00
Marketing				400.00
Tax				241.00
Profit				219.00
Data encoded [MB]	1.51	15.75	5.80	1.30
Consumer Cost [$]	6000.00	64680.00	35204.00	2647.17
Storage Cost [$/MB]	3973.73	4106.67	6074.89	2036.29

Equipment cost for our synthesis machine were roughly 100.000 US$. Assuming the annual capital expenses (CAPEX) are about four times as much[18], which includes the time value of money and a conservative depreciation over 10 years. A machine that is running 300 syntheses every year therefore costs approximately 1333 US$/synthesis. A detailed cost rundown for the chemicals needed in a synthesis can be found in

Supplementary Table 2. For the personnel, 4 h/synthesis including quality control at 50 US$ h^{-1} and a conservative 100% overhead cost for electricity, buildings, facilities, etc, were estimated. Electricity costs for the DNA synthesis are included here. This cost was copied 1:1 for marketing expenses. A universal VAT (value added tax) of 10% and a 10% profit margin were included. For the experiment with a data load of 1.3 MB which can still be scaled to having a net density of ~0.3 bits nt^{-1} on the same machine, a price of 2036 US$ MB^{-1} can be calculated. This shows that even with conservative cost calculations the synthesis method employed in this work is already superior by a factor of ~2. Taking into consideration the development status and great scaling potential, considerable improvements can be achieved

Supplementary Methods

Monte Carlo simulations

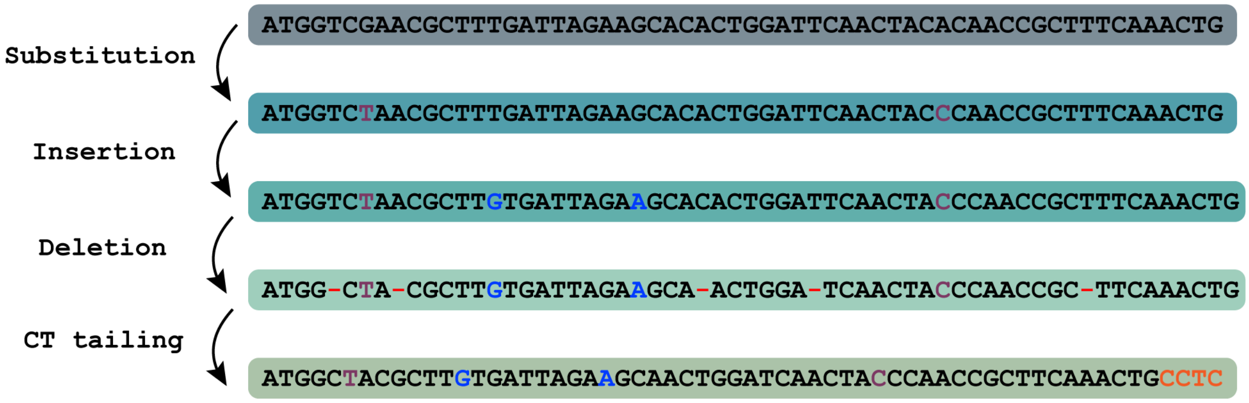

Supplementary Fig. 6: **Workflow for the Monte Carlo type simulations**. Starting from the original sequence (grey), three subsequent Monte Carlo experiments are conducted. Eventually a CT tail is appended.

Supplementary Fig. 6 illustrates the simulation sequence. The untreated design file, that has 16343 sequences with a length of 60 characters each, was taken as input. In the first step, a randomly generated number of substitutions with a Poisson distributed probability λ (poissrnd(), Matlab), that represents the average number of occurrences, was generated and fed to a random number permeator (randperm(), Matlab) which chooses λ numbers between 1 and 60. These represent the indices which are replaced by another randomly generated instance of A, C, G, or T (randseq(), Matlab). Consequently, the characters with these indices were substituted.

To simulate insertion errors, randomly generated bases were inserted after the index position of a second set of Poisson distributed random numbers between 1 and 60, for each sequence.

In the consecutive step, deletion errors were introduced. In order to achieve the deletion of positions, the corresponding characters of the randomly selected indices were simply erased from the array. The described operations were conducted with Matlab Version 2018a.

CT tailing was done with Python 3.8. C and T tails, were randomly generated and concatenated to every sequence. The probability of C and T were set to ~85% and ~15% respectively, corresponding to observed values from sequencing data analysis. Every sequence was sliced after position 60 and consequently represents a simulated erroneous feature.

Supplementary References

1. Makarov, V. & Kurihara, L. Methods and Composition for Size-Controlled Homopolymer Tailing of Substrate Polynucleotides by Nucleic Acid Polymerase. (2018).
2. Haveliwala, T. H., Gionis, A. & Indyk, P. Scalable Techniques for Clustering the Web. *Third International Workshop on the Web and Databases (WebDB 2000)* 6 (2000).
3. Rashtchian, C. *et al.* Clustering Billions of Reads for DNA Data Storage. *Advances in Neural Information Processing Systems* **2017-December**, 3361–3372 (2017).
4. Broder, A. Z. On the resemblance and containment of documents. in *Proceedings. Compression and Complexity of SEQUENCES 1997 (Cat. No.97TB100171)* 21–29 (IEEE Comput. Soc, 1998). doi:10.1109/SEQUEN.1997.666900.
5. Blanchard, A. P., Kaiser, R. J. & Hood, L. E. High-density oligonucleotide arrays. *Biosensors and Bioelectronics* **11**, 687–690 (1996).
6. Lefkowitz, S. M., Fulcrand, G., Dellinger, D. J. & Hotz, C. Z. Functionalization of substrate surfaces with silane mixtures. (2001).
7. Indermuhle, P. F., Marsh, E. P., Fernandez, A., Banyai, W. & Peck, B. J. Methods and devices for de novo oligonucleic acid assembly. (2016).
8. Dill, K., Montgomery, D. D., Wang, W. & Tsai, J. C. Antigen detection using microelectrode array microchips. *Analytica Chimica Acta* **444**, 69–78 (2001).
9. *Microarrays: preparation, microfluidics, detection methods, and biological applications*. (Springer, 2009).

10. Hornbeck, L. J. Digital Light Processing and MEMS: reflecting the digital display needs of the networked society. in (ed. Parriaux, O. M.) 2–13 (1996). doi:10.1117/12.248477.
11. Church, G. M., Gao, Y. & Kosuri, S. Next-generation digital information storage in DNA. *Science* 1226355 (2012).
12. Goldman, N. *et al.* Towards practical, high-capacity, low-maintenance information storage in synthesized DNA. *Nature* **494**, 77–80 (2013).
13. Grass, R. N., Heckel, R., Puddu, M., Paunescu, D. & Stark, W. J. Robust Chemical Preservation of Digital Information on DNA in Silica with Error-Correcting Codes. *Angewandte Chemie International Edition* **54**, 2552–2555 (2015).
14. Erlich, Y. & Zielinski, D. DNA Fountain enables a robust and efficient storage architecture. 5 (2017).
15. Organick, L. *et al.* Random access in large-scale DNA data storage. *Nature Biotechnology* **36**, 242–248 (2018).
16. Lee, H. H., Kalhor, R., Goela, N., Bolot, J. & Church, G. M. Enzymatic DNA synthesis for digital information storage. *bioRxiv* (2018) doi:10.1101/348987.
17. *FUJIFILM Dimatix Collaborates with Agilent in Developing Inkjet Technology for Advanced Life Sciences Applications*. https://www.nanowerk.com/news/newsid=4865.php (2008).
18. Cussler, E. L. & Moggridge, G. D. *Chemical Product Design*. (Cambridge University Press, 2011). doi:10.1017/CBO9781139035132.
